# Observation of Topological Chirality Switching Induced Freezing of a Skyrmion Crystal

**DOI:** 10.1002/adma.202513067

**Published:** 2025-10-28

**Authors:** John Fullerton, Yue Li, Harshvardhan Solanki, Sergey Grebenchuk, Magdalena Grzeszczyk, Zhaolong Chen, Makars Šiškins, Kostya S. Novoselov, Maciej Koperski, Elton J. G. Santos, Charudatta Phatak

**Affiliations:** ^1^ Materials Science Division Argonne National Laboratory Lemont IL 60439 USA; ^2^ Institute for Condensed Matter and Complex Systems School of Physics and Astronomy University of Edinburgh Edinburgh EH9 3FD UK; ^3^ Institute For Functional Intelligent Materials National University of Singapore Singapore 117544 Singapore; ^4^ Department of Materials Science and Engineering National University of Singapore Singapore 117544 Singapore; ^5^ School of Advanced Materials Peking University Shenzhen Graduate School Peking University Shenzhen Guangzhou 518055 China; ^6^ Donostia International Physics Center Donostia‐San Sebastian 20018 Spain; ^7^ Materials Science and Engineering Department Northwestern University Evanston IL USA

**Keywords:** 2D phase transition, chirality, lorentz microscopy, skyrmionic bubbles, van der Waals materials

## Abstract

Magnetic skyrmions are topologically protected quasi‐particles with a well‐defined chirality. Control over their chirality is proposed as an additional feature for encoding data bits or as qubits in quantum computing due to their high efficiency and stability against achiral magnetic textures. Here it is shown that an in‐plane magnetic field can be utilized to reshape the energy barriers between different skyrmionic bubbles (e.g., Bloch type, type‐II) enabling spontaneous chirality fluctuations with a frequency that increases with the strength of the in‐plane field. The insulating van der Waals ferromagnet CrBr_3_ is used as an archetypal system for low damping, reduced energy dissipation and a high number of magnetic phases to capture the chirality dynamics in real time through cryo‐Lorentz transmission electron microscopy. It is observed that the interplay between the intrinsic Dzyaloshinskii–Moriya interaction and out‐of‐plane field biased the chirality dynamics, favoring one handedness over the other. A remarkable consequence of the spontaneous chirality switching mechanism is that it induces a freezing (or crystallization) process in the skyrmion lattice. As the bubbles fluctuate between Bloch and type‐II they elongate and shrink parallel to the in‐plane field. Subsequently, the overall lattice crystallizes along the in‐plane field direction, inducing a phase transition from a disordered liquid state to a hexatic phase where skyrmions are highly ordered resembling that of a solid. The results indicate chirality as an active element in the creation of topologically protected skyrmion crystals unveiling pathways toward chiral spintronic device platforms with tunable embedded configuration.

## Graphical abstract

Using Lorentz Transmission electron microscopy, it is shown that in the insulating van der Waals ferromagnet, CrBr_3_, a magnetic field can cause Bloch skyrmionic bubbles to spontaneously switch their chirality. As achiral type‐II bubbles are an intermediate state, the bubbles rapidly elongate and shrink when switching, thereby inducing a freezing of the overall bubble lattice. The control over skyrmion chirality and order shown here can allow the creation of designer skyrmion crystals for utilization in unconventional computing.

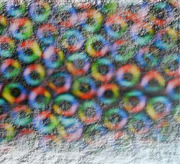

## 1 Introduction

The chirality of a system defines whether it is super‐imposable on its mirror image.^[^ ^1^, ^2^, ^3^ ^]^ Chirality plays an important role in many areas of science, including drug effectiveness, chemistry, and magnetism.^[^ ^4^, ^5^, ^6^, ^7^ ^]^ Magnetic skyrmions are topologically protected quasi‐particles with a well‐defined chirality^[^ ^8^, ^9^, ^10^ ^]^ that are of fundamental interest due to their topology and complex dynamics, as well as having potential applications in quantum information sciences and unconventional computing.^[^ ^11^, ^12^, ^13^, ^14^ ^]^ Skyrmion bubbles are typically stabilized by the Dzyaloshinskii‐Moriya interaction (DMI, an antisymmetric exchange energy which induces a chiral spin rotation), or long‐range dipolar interactions. The dynamics of skyrmions have shown promise for Brownian, stochastic and neuromorphic computing and skyrmion chirality has been proposed as qubits for quantum computing.^[^ ^15^, ^16^, ^17^, ^18^, ^19^ ^]^ Hence, improving the understanding and control over skyrmion chirality and motion is essential for developing spintronic devices with additional functionalities.^[^ ^20^, ^21^, ^22^, ^23^, ^24^, ^25^ ^]^ Additionally, extended skyrmion lattices offer a unique platform for expanding our fundamental understanding of ordering behavior, phase transitions and topological defect dynamics in 2D systems due to a high degree of flexibility.^[^ ^26^, ^27^, ^28^, ^29^, ^30^, ^31^ ^]^

2D van der Waals (vdW) materials have emerged in recent years as a unique material platform for controlling and stabilizing topological spin textures through varying composition, thickness and external excitations.^[^ ^10^, ^32^, ^33^, ^34^, ^35^, ^36^ ^]^ The insulating vdW CrBr_3_ ferromagnet has recently been shown to possess a rich phase diagram involving multiple possible magnetic textures. Consequently, it is possible to reliably generate Bloch skyrmion lattices of mixed chirality in CrBr_3_ by cooling the sample through its Curie temperature (≈37 K) with an out‐of‐plane field applied.^[^ ^32^ ^]^ CrBr_3_ crystallizes in a trigonal $R\bar{3}$ space group, with a hexagonal arrangement of Cr atoms and lattice parameters as follows: a = 6.38 Å, b = 6.38 Å, and c = 18.95 Å.^[^ ^37^, ^38^, ^39^ ^]^ While the symmetry of the space group allows for a weak DMI arising from next‐nearest‐neighbor interactions^[^ ^40^, ^41^, ^42^, ^43^ ^]^ the formation of Bloch skyrmions is primarily facilitated by dipolar interactions. Such a feature opens the prospects of chiral topological matter in ultrathin magnets, which interfacial engineering can be used to design sophisticated device applications via vdW technologies. However, no apparent control on how the different chiralities behave or interact with each other to result in a skyrmion crystal has been reported so far. Similar issues are also observed in other magnets, not necessarily vdW type.^[^ ^44^, ^45^ ^]^

Here, we used cryo‐Lorentz transmission electron microscopy (LTEM) to image the real time dynamics of skyrmionic bubbles in CrBr_3_. We found that the application of an in‐plane magnetic field transforms the Bloch skyrmionic bubbles into topologically trivial type‐II bubbles, significantly altering the energy landscape of the system.^[^ ^46^, ^47^, ^48^, ^49^ ^]^ By optimizing the strength of the in‐plane field, we can substantially minimize the energy barrier between skyrmionic bubbles of opposing chirality (right‐hand (RH), left hand (LH)). This induces spontaneous and reversible chirality switching of the spin textures, assisted by random thermal fluctuations and with achiral type‐II bubbles as an intermediate step in the transition, where the rate of chirality switching increases rapidly with the in‐plane magnetic field. We observed that a weak DMI breaks the degeneracy between the Bloch chirality of the bubbles hence leading to an increasing population of a desired chirality depending on the direction of the out‐of‐plane magnetic field. Therefore allowing us to control both the frequency of chirality switching and the relative populations of LH and RH skyrmionic bubbles. Additionally, we show that an in‐plane field speeds up the relaxation and diffusion of topological defects in the skyrmion lattice, thus inducing a phase transition from a liquid to a hexatic state. We noticed that this crystallization phenomenon arises as a consequence of the elongation of the bubbles as they transition through a type‐II bubble state while switching chirality, providing a strong link between individual chirality fluctuations and collective skyrmion behavior. The complex, time‐resolved skyrmionic dynamics displayed here shows great promise for unconventional computing applications and for advancing the fundamental understanding of how to control skyrmionic bubble chirality lattice dynamics in quantum materials.

## 2 Results

**Figure 1:**
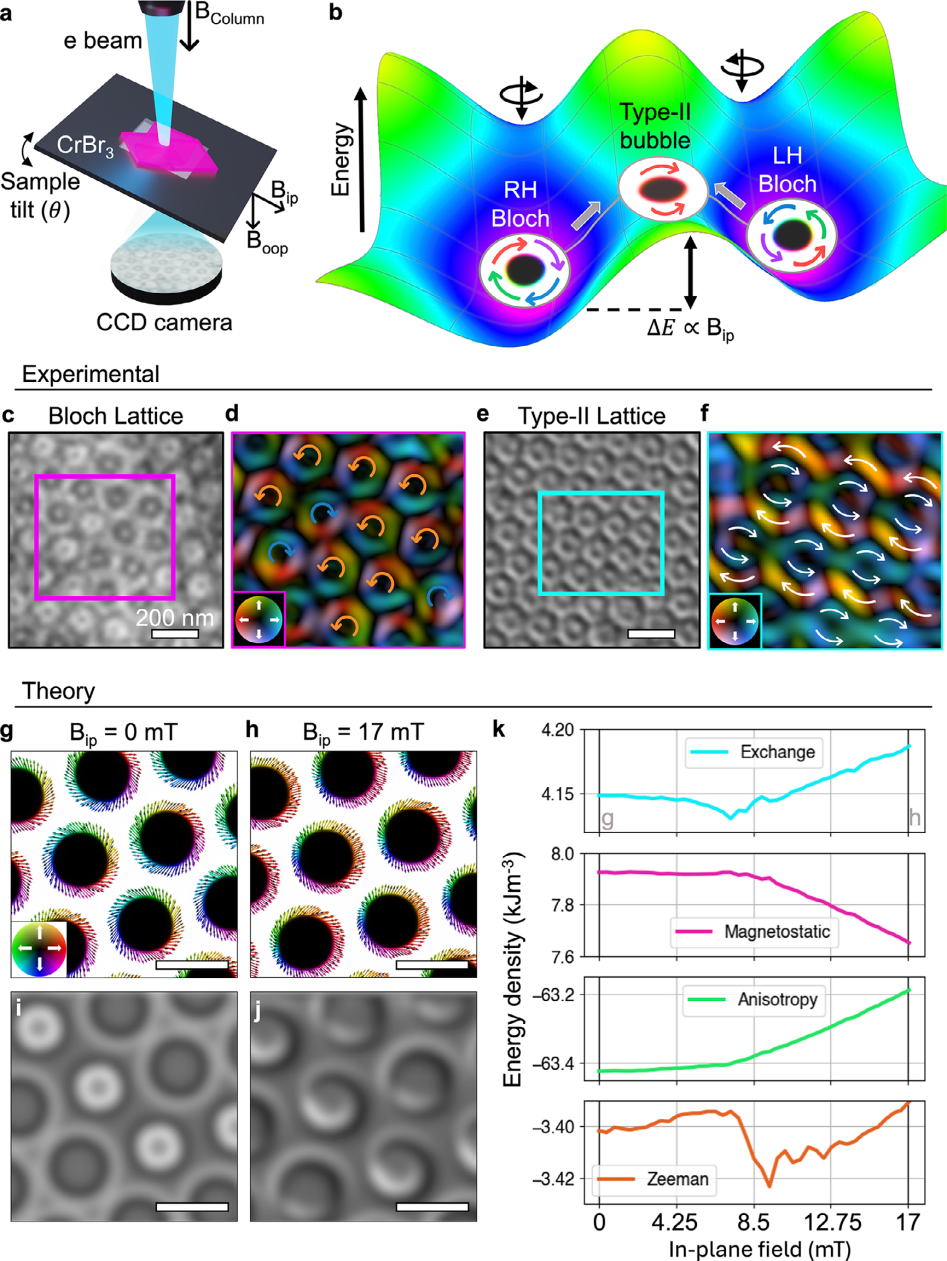
Manipulating the energy landscape of CrBr_3_ with in‐plane magnetic fields. a) Schematic depiction of experimental LTEM setup, where tilting the sample relative to the electron beam produces an in‐plane component of the magnetic field (B_*ip*_). b) Schematic of the energy landscape of the system, with Bloch skyrmionic bubbles as the ground state and an energy barrier (ΔE) between type‐II bubbles and opposing chiralities. c) Experimental LTEM image of a mixed Bloch skyrmionic bubble lattice at 13 K, stabilized by field cooling at B_*oop*_ = 30 mT. d) Reconstruction of the magnetic induction of blue box in part (c) showing the mixed chiralities of skyrmionic bubbles. e) Tilting the sample in LTEM creates an in‐plane field component which creates a lattice of type‐II bubbles. f) Reconstruction of the magnetic induction of pink box in part (f) showing alignment of domain walls in the type‐II bubbles. g–h) Micromagnetic simulations of a Bloch skyrmionic bubble lattice at *B*_*ip*_ = 0 mT (g) and at *B*_*ip*_ = 17 mT (h), with B_*oop*_ = 500 G. The scale bar is 200 nm. i‐j) Simulated LTEM images of the simulations in parts g and h, respectively. k) Micromagnetic energies as a function of *B*_*ip*_, starting and ending at the states shown in parts (g) and (h), respectively.

In CrBr_3_, distinct topological states can be stabilized by field cooling with an out‐of‐plane magnetic field (**Figure** **1**a).^[^ ^32^ ^]^ Here, we field‐cooled a ≈100 nm thick exfoliated flake of CrBr_3_ (Figure S1, Supporting Information) at 30 mT in order to create a disordered lattice of mixed chirality Bloch skyrmions at 13 K (Figure 1c). The out‐of‐plane magnetic field (B_*oop*_) provided by the objective lens of the TEM stabilizes both RH (black in LTEM image) and LH (white) Bloch skyrmionic bubbles, which are the two possible equilibrium states (Figure 1b; Figure S2, Supporting Information). The handedness in this case is given by the core direction (out‐of‐the‐plane) and the circulation of the spins at the perimeter of the bubbles. To image the spin circulation, we reconstruct the in‐plane magnetic induction using a single image transport of intensity equation (SITIE) method,^[^ ^50^, ^51^ ^]^ which clearly reveals the mixed distribution of RH and LH Bloch skyrmionic bubbles (Figure 1d).^[^ ^10^, ^32^, ^50^ ^]^ Tilting the sample in our LTEM setup we can introduce an in‐plane component of the magnetic field (*B*_*ip*_), which facilitates a transformation from Bloch skyrmions into topologically trivial type‐II bubbles (Figure 1e; Figure S3, Supporting Information). By reconstructing the magnetic induction maps, we show that the domain walls of the type‐II textures are reorientated to point along the in‐plane field direction (Figure 1f).^[^ ^10^, ^50^ ^]^ The controllable transition between topologically protected Bloch skyrmions and topologically trivial type‐II bubbles shows how the energy landscape of the system can be altered by an in‐plane magnetic field, as shown schematically in Figure 1b. When the magnetic field is purely out‐of‐plane, we stabilize Bloch skyrmions with an energy barrier (ΔE) between opposing chiralities and the type‐II state at the saddle point. This energy barrier is directly related to the strength of the in‐plane field and can be overcome to transform the topology of the bubble lattice. As the in‐plane field is increased, the relative energy of the Bloch bubbles increases as they are destabilized. Subsequently, the type‐II bubbles become the most favorable state above a critical in‐plane field value (Figures S2 and S4, Supporting Information show further schematics of the effect of an in‐plane field on the magnetic energy landscape). One implication of tuning the energy barrier between states in this way is that random thermal fluctuations might cause spontaneous switching of skyrmion chiralities^[^ ^23^, ^48^, ^52^, ^53^ ^]^ once the barrier is fully negligible (Figure S4, Supporting Information). Indeed, this process has been captured in our measurements, as discussed in the following.

To gain further understanding of the energy landscape, we explore the transition from Bloch skyrmions to type‐II bubbles through micromagnetic simulations (Figure 1g–k see Experimental Section and Section S3, Supporting Information for details). We start by stabilizing a lattice of mixed chirality Bloch skyrmions at a B_*oop*_ = 50 mT (the layer integrated magnetization is shown in Figure 1g). By systematically increasing the in‐plane field component, we observe the transition toward type‐II bubbles as *B*_*ip*_ increases to 17 mT (Figure 1h). Simulated LTEM images from these simulations are included in Figure 1i,j, respectively, as a comparison to the experimental images in Figure 1c,e. As the magnetization evolves from Bloch bubbles toward type‐II bubbles, we tracked the evolution of the exchange, magnetostatic, anisotropy and Zeeman energy density terms as a function of *B*_*ip*_ (Figure 1k).^[^ ^54^ ^]^ We see that the transition toward type‐II bubbles begins at *B*_*ip*_ ≈ 7 mT and is largely driven by a notable reduction in magnetostatic energy until it reached the final field at 17 mT. At the transition onset, there is an initial reduction in exchange and Zeeman energy densities due to the alignment of the spins in the bubbles to *B*_*ip*_, while the out‐of‐plane anisotropy energy increases significantly. This analysis demonstrated experimentally and computationally that the spin topology and magnetic energy landscapes can be controllably manipulated by an in‐plane magnetic field.

**Figure 2:**
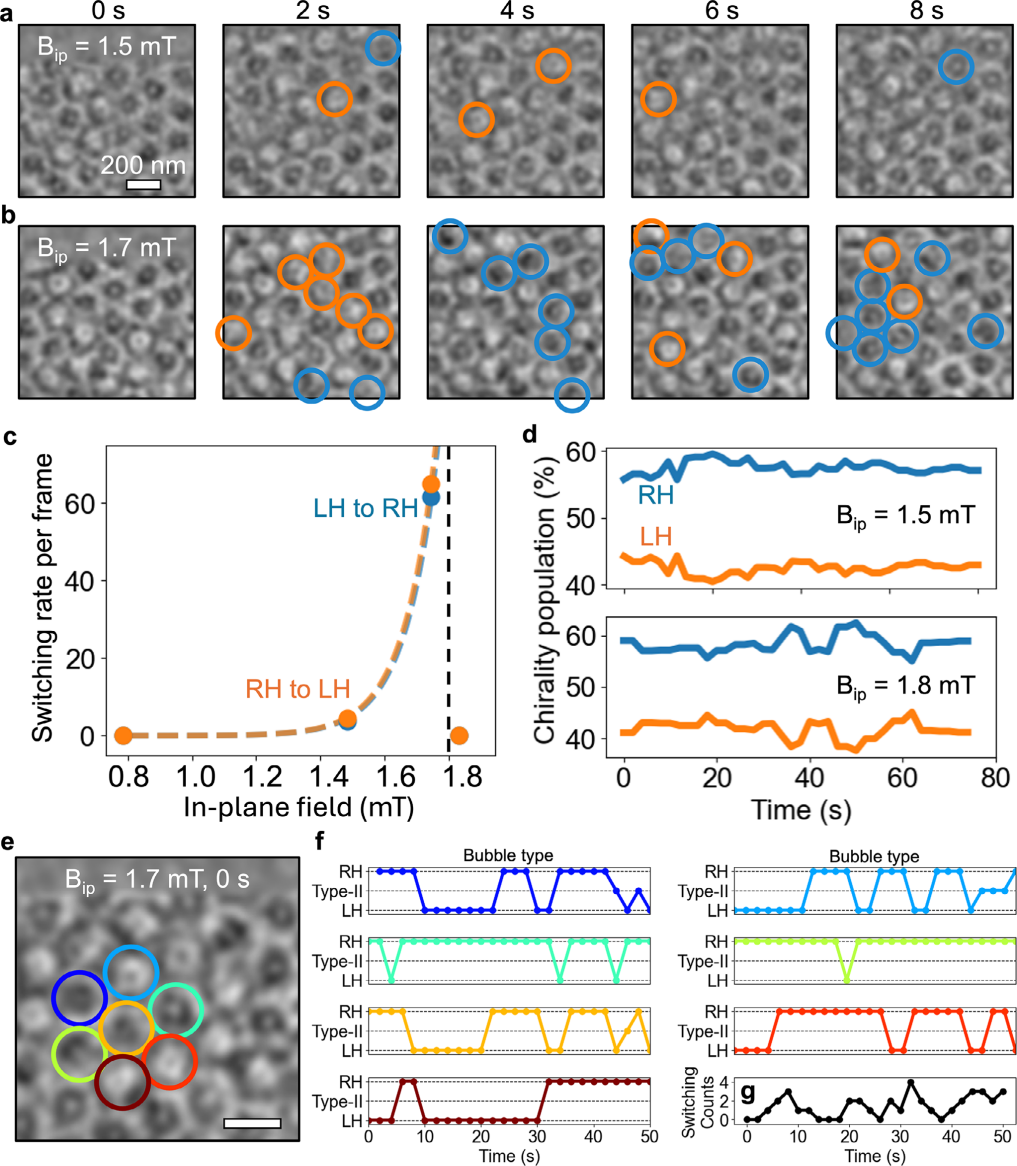
Controlling the chirality rate with in‐plane magnetic fields. a–b) Time series of LTEM images at fields of *B*_*oop*_ = 50 mT and *B*_*ip*_ = 1.5 (a) and 1.7 mT (b). Chirality switching events are highlight by blue (LH, white bubbles, to RH, black bubbles) and orange (RH to LH) circles. c) Plot of chirality switching rate per frame against the magnitude of in‐plane magnetic field. The blue and orange dashed lines are guides to the eye, and the black dashed line indicates the field value where a uniform type‐II lattice is formed. d) Plots of the populations of skyrmionic bubble chiralities over time for *B*_*ip*_ = 1.5 (top) and 1.7 mT (bottom), respectively. e–g) The spontaneous switching events of a cluster of 7 skyrmionic bubbles (e) are plotted as a function of time (f). g) The number of switching events for the seven skyrmionic bubbles at each timestep.

Several important questions arise from these results such as how the different chiralities change in situ at a given in‐plane field. We noticed that by changing the magnetic field strength we can control the rate of thermal switching between magnetic states. In this case, we maintain a constant temperature (13 K) and *B*_*oop*_ = 50 mT to isolate the effect of the in‐plane magnetic field. In **Figure** **2**a,b, we show two series of LTEM images of a 1 × 1 µm^2^ area over a period of 8s with increasing in‐plane field strength, respectively. For *B*_*ip*_ = 1.5 mT (Figure 2a), we observed six bubbles spontaneously switching chirality over the 8 s time period. These chirality switches are highlighted by blue and orange circles for the LH to RH and RH to LH transitions, respectively. When the field is increased to *B*_*ip*_ = 1.7 mT (Figure 2b) we detected 30 chirality switching events, showing a significant increase in switching with field strength. We also direct the reader to Videos S1–S5 and S7 (Supporting Information), which show these spontaneous chirality switching events for multiple external field values over longer time periods. In Figure 2c, we average the number of switching events per 2s frame over a larger series of 5 × 5 µm^2^ images as a function of the in‐plane magnetic field. We clearly see a rapid increase in switching events with the field until a critical value of ≃1.7 mT where stable type‐II bubbles are formed (e.g., Figure 1e). This spontaneous chirality switch is due to random thermal fluctuations in the material.^[^ ^14^, ^23^, ^47^, ^48^ ^]^ As CrBr_3_ is an insulator, it can be expected that there will be charging effects under electron beam irradiation. While it is difficult to fully rule out charging effects, in our measurements we coat the sample with platinum which we believe reduces the effect of the electron beam considerably. Hence that the behavior we observe are mainly driven by thermal fluctuations and the application of a magnetic field. Although the measurement temperature is at the minimum attainable temperature of the liquid helium TEM stage (13 K), where thermal noise is incidentally small, the field‐reduced magnetic energy barriers enable this chirality toggle switching at will. It should be noted that there might be switching events missed in our analysis due to the time resolution per frame used (2 s), which suggested that considerably more chirality switching might be ongoing in the samples. However, the underlying picture remains the same with the ability to controllably induce skyrmionic chirality switching through the application of an in‐plane magnetic field.

Interestingly, while this spontaneous chirality switching is occurring, we can also track the relative populations of RH and LH skyrmions over time. In Figure 2d, we plot how the percentage of skyrmionic populations (RH – blue, LH – orange) vary over time for in‐plane fields of different magnitudes. In both cases, we see uneven chirality populations between RH and LH skyrmions (≈57–43.% at 1.5 mT; and ≈60–40% at 1.7 mT). These values are also comparable with the relative chirality populations seen after field cooling (and after removing an applied in‐plane field from a type‐II lattice in this material, see Figure S3, Supporting Information) and are consistent across several samples.

To ascertain whether the skyrmionic chirality switching discussed above is inherently an individual or collective behavior, we track the switching events of a cluster of seven bubbles over a longer period of time (Figure 2e,f). We initially show each magnetic object (RH, LH or type‐II) considered and follow the number of switching events seen in each frame. In this case, we don't observe any spatial or temporal correlation in the chirality switching. We also evaluate the chirality switching in Section S2.3 (Supporting Information), using the pairwise spin correlation function which supports the observation that these are random, individual switching events. The random fluctuations in skyrmion chirality unveiled here further emphasize the potential of topological textures for stochastic computing applications, allowing future devices to be based on isolated bubbles or extended lattices.

**Figure 3:**
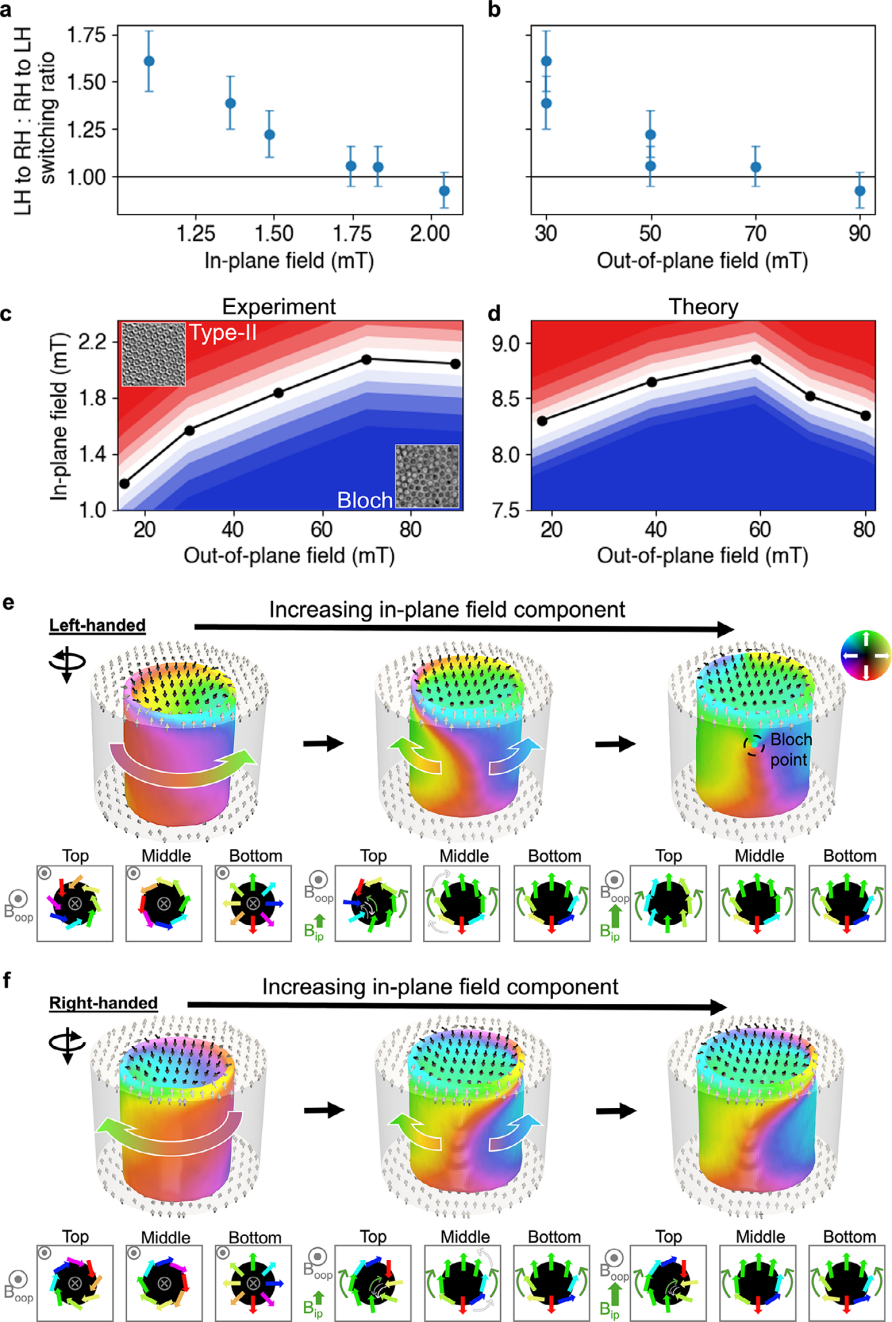
Preferential Bloch chirality switching. a,b) Plots of the ratio of LH to RH and RH to LH switching events against the in‐plane *B*_ip_ (a) and out‐of‐plane *B*_opp_ (b) magnetic field components. c,d) Phase diagrams of *B*_ip_ versus *B*_oop_ for the transition from Bloch to type‐II bubbles in experiments and theory, respectively. The white region around the black dots corresponds to the stochasticity region where small variations of magnetic fields might affect the creation of either type of bubbles. e,f) 3D micromagnetic simulations and schematics of the transition of LH (e) and RH (f) skyrmions towards type‐II bubbles with increasing *B*_ip_ (and reducing B_oop_) from left to right. The LH bubble switches to a type‐II bubble at lower field values than the RH due to symmetry breaking from the DMI and the torque from the B_oop_. Here, the top surface switches to a type‐II bubble forming a Bloch point within the film (black dashed circle, right in Figure 3e). The schematics show the top, middle and bottom surface of the skyrmionic bubbles where the curved green arrows represent the Zeeman energy applied by *B*_ip_, the white arrows represent the DMI contribution, and the gray arrows represent the rotational torque from the B_oop_.

As discussed above (Figure 2d), we consistently found a higher population of RH skyrmions compared to that in LH orientation. Subsequently, we also observe an inhomogeneity in the ratio of switching events at certain field values. In **Figure** **3**a,b, we plot the ratio of LH to RH, and RH to LH switching events against the in‐plane (Figure 3a) and out‐of‐plane (Figure 3b) magnetic fields. For larger values of both field projections, we see that the ratio of switching events tend towards 1:1. Hence, for larger fields we would expect the relative population of bubble chiralities across an extended lattice to stay consistent while these random switching events occur. However, for lower field magnitudes there is a rise in the chirality switching ratio, which would lead to an increase in the population of RH skyrmionic bubbles and a reduction in the LH population over time. In Figure 3c,d, we plot from both experiments and theoretical modeling, the critical in‐plane and out‐of‐plane field values for transforming a Bloch skyrmion lattice (blue) into a type‐II bubble lattice (red). The larger magnitudes of the in‐plane fields in the theory plot relative to the experiments are due to slight differences in the magnetic parameters used in the calculations and a much simpler modeling setup relative to the LTEM protocol. The simulations do not consider any external components (e.g., substrates, etc.) that might affect the magnitude of the final field applied to the samples. However, the agreement between measurements and modeling remains sound. Hence, in both cases we see that for increasing out‐of‐plane field, we require a larger in‐plane field to induce the transition to type‐II bubbles.

With increasing out‐of‐plane field, the skyrmion diameter decreases significantly (Figure S8, Supporting Information). Consequently, this shrinks the length of the domain walls (i.e., the circumference of the skyrmionic bubbles), thereby requiring more energy (field) to induce a transformation to type‐II bubbles. Hence, this bubble shrinking likely reduces the domain wall energy of the bubbles and thereby explains why the chirality switching ratio tends to a 1:1 relation with increasing out‐of‐plane field.

To explain the uneven chirality switching ratio observed at lower applied fields, we investigate its mechanism through micromagnetic modeling (Figure 3e,f). In our system, the skyrmionic bubbles are primarily stabilized by dipolar interactions. Hence, we observe hybrid chiral skyrmionic bubbles across the thickness of the sample with a Bloch component in the center and Néel caps at the top and bottom surfaces (e.g., in this continuous limit, the textures resemble tubes throughout the layers). However, in CrBr_3_, there exists a weak DMI^[^ ^32^ ^]^ which, unlike other vdW systems, doesn't dominate the system by creating Néel skyrmions.^[^ ^24^, ^55^, ^56^ ^]^ Instead, this weak DMI works to expand the bottom Néel cap, shrinks the top cap, and shifts the central Bloch component upwards (highlighted in schematics for Figure 3e,f). Hence, we see a complex z‐dependent, 3D profile of the Bloch skyrmionic bubbles which significantly impacts the chirality switching process. As an in‐plane field is applied (left‐to‐right in Figure 3e,f) the spins in the bottom and middle layers of the skyrmionic bubbles rotate outward to align towards the applied field. This outward rotation is also assisted by the DMI present in the sample and is the same for both the LH and RH skyrmionic bubbles. Nevertheless, there is a difference in the top layers that depends on the initial Bloch chirality. On the top layer, some spins begin to rotate inwards towards the applied field (left‐hand side for LH chirality and right‐hand side for RH). However, this inward rotation is opposed by the DMI energy in the sample and would require an increased in‐plane field component to facilitate this rotation. What breaks the symmetry in this case is a combination of the DMI energy and a rotational torque provided by the out‐of‐plane field component.^[^ ^57^, ^58^ ^]^ Due to the out‐of‐plane field, there is a right‐handed rotational torque experienced by the spins, which assists the chirality switching for the LH skyrmions, but opposes it for the RH ones (see curly gray arrows in the top‐layer schematics). Hence, the LH skyrmionic bubble switches to a type‐II bubble, through the injection of a Bloch point from the top layer, at field values lower than those of the RH skyrmions.^[^ ^59^ ^]^ Correspondingly, this effect also impacts the relative populations of skyrmionic bubble chirality after field cooling. This explains why in our experimental setup we consistently observe a preference for RH skyrmions.

In Figure S9 (Supporting Information), we include simulations where no DMI is present which show that LH and RH skyrmions become type‐II bubbles at the same value of the in‐plane field. We also note that reversing the direction of the out‐of‐plane field (or sign of the DMI) should create the opposite effect (i.e., favor switching of the RH skyrmionic bubble). Furthermore, this explains the trends shown in the chirality switching ratios (Figure 3a). As the chirality switching events are induced by thermal fluctuations, a larger in‐plane field means that the thermal energy is sufficient to overcome the energy barriers for both LH and RH skyrmionic bubbles. Hence, the chirality switching ratio tends to 1:1. However, at lower in‐plane fields, the thermal energy more easily overcomes the energy barrier for switching the LH skyrmionic bubble (in this case). By controlling the strength of the in‐plane field and the direction of the out‐of‐plane magnetic field, we can favor either a RH to LH or a LH to RH switching pathway for the skyrmionic bubbles. Therefore, the DMI‐induced field‐driven symmetry‐breaking effect described here gives us a pathway to controllably tune the relative populations of LH and RH skyrmions and allows a route towards stabilizing a homochiral skyrmion lattice in CrBr_3_.

**Figure 4:**
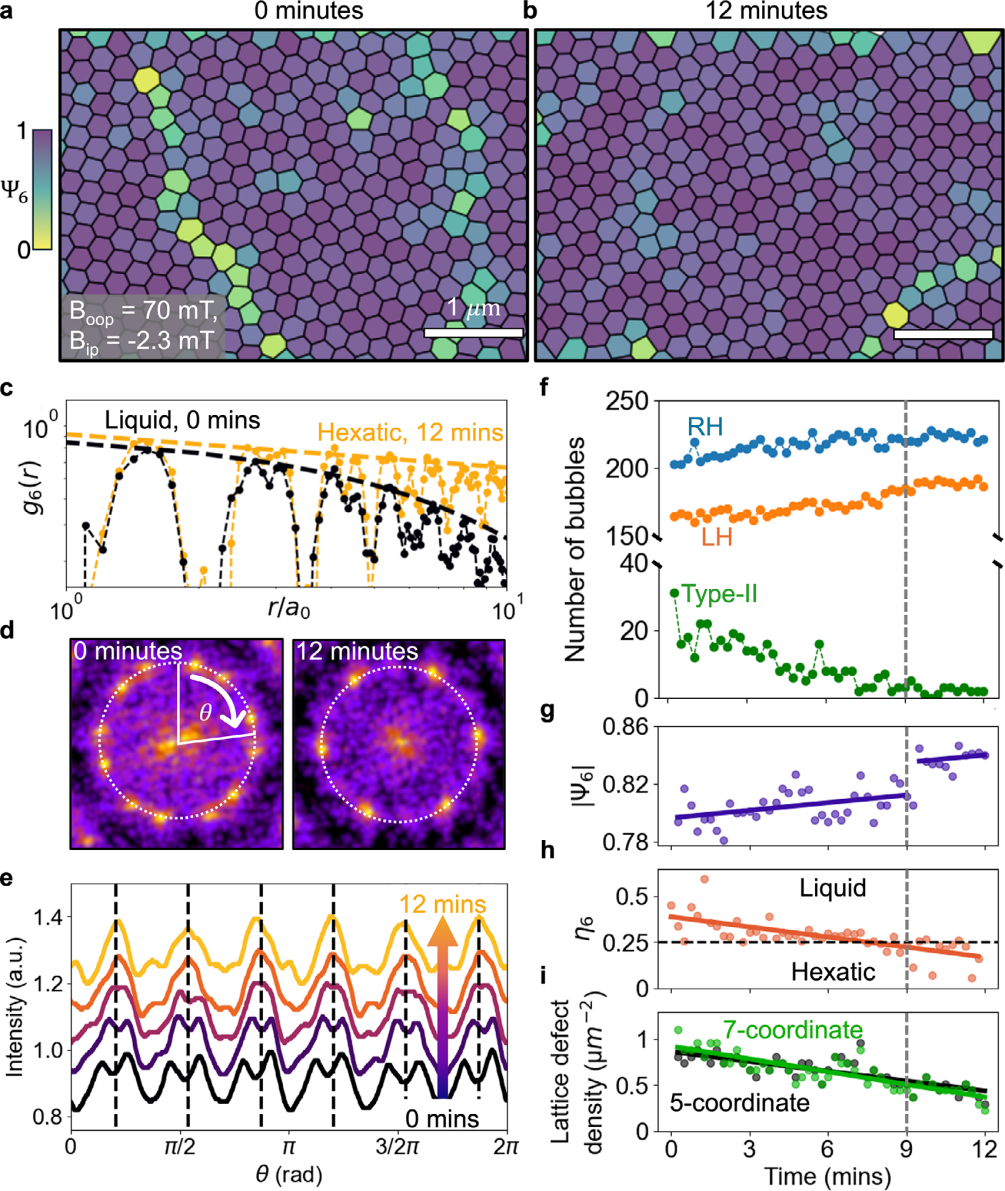
Liquid to hexatic skyrmion lattice phase transition driven by chirality switching. a,b) Voronoi tessellations of a Bloch skyrmionic bubble lattice after field cooling (a) and 12 min later (b) with a magnetic field of B_oop_ = 70 and *B*_ip_ = −2.3 mT. c) Orientational correlation function for the skyrmionic bubble lattices at 0 (black) and 12 (yellow) minutes, calculated from the lattices shown in (a) and (b), respectively. d) FFTs calculated from the lattices shown in (a) and (b), respectively, where θ is defined as the azimuthal angle. e) Plots of the lattice peak intensity vs θ calculated from FFT's of the skyrmion lattices over time. f–i) Plots of the number of each bubble type (f), Ψ_6_ parameter (g), the power law exponent η_6_ (h), and lattice defect density (i) over time.

The introduction of an in‐plane field can not only create order through chirality switching events, but it can also help to drive the diffusion of lattice defects. In **Figure** **4**, we describe the evolution of a skyrmion lattice over time at a given applied field (Figure 4a,b). We initially field‐cooled the sample at B_oop_ = 30 mT (no tilting), then the field is subsequently increased to (B_oop_ = 700 G) and the sample was tilted such that *B*_ip_ = −2.3 mT. Figure 4a,b show Voronoi tessellations of a Bloch skyrmion lattice immediately after increasing the field (Figure 4a) and 12 min later (Figure 4b) while the field and temperature are kept constant at *B*_*oop*_ = 70 mT, *B*_*ip*_ = −2.3 mT and 13 K, respectively. The tessellations are colored by the hexatic order parameter Ψ_6_(*r*) described below:

(1) $$\Psi_{6}\left(r\right)=\frac{1}{N_{m}}\sum_{j=1}^{N_{m}}e^{-6i\theta_{j}}$$

where *N*_*m*_ is the number of nearest neighbors for each position *r*, and θ_*j*_ is the angle between the bond to each nearest neighbor (*j*) at fixed axis.^[^ ^27^, ^28^, ^29^ ^]^ The hexatic ordering parameter tends to 1 (purple) for a perfect hexagonal lattice and decreases (green to yellow) for more disordered sites. As such, we observe grains of ordered skyrmions (purple) separated by grain boundaries consisting of multiple lattice defects, e.g., 5‐ and 7‐coordinate skyrmions (Figure 4a). After 12 min, we see a significant reduction in the number of lattice defects and a more uniform hexagonal lattice (Figure 4b) with Ψ_6_(*r*) ≈1 almost entirely over the surface (Video S7, Supporting Information shows the time evolution of the LTEM images and Video S8 (Supporting Information) shows the time evolution of the Voronoi tessellations).

We can verify the progression in lattice ordering and the 2D phase by calculating the orientational correlation function:

(2) $$g_{6}\left(r\right)=\frac{1}{N_{r}}\sum_{i,j}^{N_{r}}\Psi_{6}\left(r_{i}\right)\Psi_{6}^{*}\left(r_{j}\right)$$

where *N*_*r*_ is the number of site distances *r* apart.^[^ ^27^, ^29^ ^]^ In Figure 4c shows *g*_6_(*r*) at the start and end of the time series (i.e., calculated from the lattices shown in Figure 4a,b, respectively). The upper envelope of each curve is fitted by an exponential decay or a power law (dashed lines). For the initial state (black line, Figure 4c), we observe an exponential decay as $g_{6}\left(r\right)\propto e^{\frac{-r}{\xi_{6}}}$, where ξ_6_ is the correlation length. Hence, we begin in the liquid phase, with no short‐ or long‐range order. At the end of the time series (yellow line, Figure 4c), a clear difference is noticed, as the *g*_6_(*r*) function now shows an algebraic decay ($g_{6}\left(r\right)\propto r^{-\eta_{6}}$, where η_6_ is the power law exponent) that is characteristic of a more ordered 2D phase. In the Section S2.4 (Supporting Information), we repeat the same experiment but at lower fields (B_*oop*_ = 30 mT and *B*_*ip*_ = 0.3 mT) to show that the system remains in the liquid phase over the same time period (Video S6, Supporting Information shows the time evolution of the LTEM images).

To provide a quantitative analysis, in Figure 4f–i, we track the number of each bubble type (RH, LH, Type‐II), hexatic order parameter Ψ_6_(*r*), the power law exponent η_6_, and the lattice defect density in the 12 min series, respectively. Figure 4f shows that for this magnitude of in‐plane field, we observe constant switching events between LH, RH and type‐II bubbles (see Video S7, Supporting Information) with the largest population being RH bubbles, as expected. Ψ_6_(*r*) shows an overall increase in the ordering (Figure 4g), matching the observations from the Voronoi tessellations. We also see an abrupt steep discontinuity in Ψ_6_(*r*) around the 9 min mark (vertical gray dashed line). The increase in Ψ_6_(*r*) coincides with a decrease in η_6_ (Figure 4h), which decreases below $\frac{1}{4}$, which is the critical value predicted by the Kosterlitz, Thouless, Halperin, Nelson and Young theory for the liquid‐hexatic phase transition.^[^ ^27^ ^]^ Throughout this process, the density of lattice defects (Figure 4i) decreases considerably resulting in a more ordered skyrmion distribution. The combination of these factors points out that a highly ordered spin texture state is established at these field values, which is correlated with the diffusion of skyrmions and topological lattice defects.

We can determine the driving force behind this liquid‐hexatic phase transition by analyzing the Fast Fourier transforms (FFTs) of the skyrmionic bubble lattice as a function of time. Examples are shown in the Figure 4d, and are calculated from the lattices shown in Figure 4a and Figure 4b. At 0 min, the FFT contains 12 peaks from two hexagonal lattice orientations, whereas after 12 min only 6 peaks from a single lattice grain remain. By plotting the radial intensity distribution of the FFT vs the azimuthal angle, θ, over time (Figure 4e), we observe that the initial 12 peaks merged with their nearest neighbor to form 6 peaks (black to purple to yellow). The initial 12 peaks are observed to merge into 6 around the 9 min mark (third curve from bottom to top in Figure 4e). This further supports the existence of a phase transition previously indicated at the abrupt jump in Ψ_6_(*r*) (Figure 4g). The vertical dashed lines in Figure 4e correspond to peaks from a uniform hexagonal lattice with one of its lattice planes aligned with the direction of the in‐plane field. Intriguingly, we observe that the peaks in the FFT align with this in‐plane field orientation, indicating a preferential crystallization direction. Thus, while we have both in‐plane and out‐of‐plane field components, we credit the phase transition to be in‐plane field induced as that seems to be more favorable for skyrmion reorganization. For the sake of completeness, we show in Section S2.5 (Supporting Information) that an increased out‐of‐plane magnetic field significantly decreases lattice ordering and introduces more defect sites rather than the annealing of the lattice. It should be noted that the application of a 180 mT out‐of‐plane magnetic field leads to the annihilation of the skyrmionic bubble lattice. Furthermore, we observe an anisotropic diffusion of the skyrmionic bubble lattice along the direction of the in‐plane field, as shown in Section S2.6 (Supporting Information).^[^ ^60^ ^]^ In the time series we discussed, the in‐plane field induces continuous fluctuations in chirality and bubble type. As the bubbles switch between Bloch and type‐II, they are constantly elongating and shrinking along the in‐plane field direction. Hence, these fluctuations in the shape and type of the bubbles influence the lattice orientation angle and induce defect diffusion by agitating the lattice grains and boundaries. This resulted in a more uniform skyrmion structure as demonstrated here and hence indicates that the chirality switching process, which is generated by the in‐plane field, plays an important role in the formation of a highly crystalline skyrmion lattice and dynamic phase transition behavior.

## 3 Conclusion

In summary, we have studied the effect of an in‐plane magnetic field on the formation of skyrmion crystals in exfoliated CrBr_3_. We observe that such an approach can induce spontaneous chirality switching events due to random thermal fluctuations. The strength of the in‐plane field is directly related to the energy barrier between different magnetic chiral states and leads to a rapid increase in the rate of chirality switching. There also exists an uneven ratio in the generation of skyrmions with RH chiralities being slightly more favored than that for LH textures. Lower values of in‐plane magnetic fields also appear to favor switching of chirality from LH to RH. We reasoned this through a symmetry‐breaking effect caused by the presence of a weak DMI interaction and an out‐of‐plane field component. We present an efficient method to select the relative populations of chiral skyrmions via magnetic field application. Additionally, skyrmion lattices in CrBr_3_ exhibit significant lattice reshuffling and defect motion over time. We observe that this reorganization can be significantly sped up through the application of an in‐plane magnetic field, leading to a phase transition from a 2D liquid state (after field cooling) to a hexatic state. This transition is coupled with a significant reduction in topological lattice defect sites, which has not been observed with the application of a sole out‐of‐plane field, and a constant switching of bubble type. This indicates that the chirality switching phenomenon is key for lattice crystallization. Hence, CrBr_3_ offers a promising system for the study of 2D particle dynamics including 2D phase transitions and diffusion of topological lattice defects where chirality might work as a mark or a descriptor to track the transitions. Furthermore, this work gives insight into novel pathways for achieving order in self‐assembled, active matter systems (e.g., molecules, polymers, colloids) by inducing fluctuations in the particles themselves induced by their potential chirality.

The large amount of flexibility and control demonstrated in the skyrmionic lattice of CrBr_3_ also has significant implications for unconventional computing applications due to the requirement of systems with high degrees of freedom and large dimensionality.^[^ ^61^, ^62^ ^]^ The ability to controllably alter the size, density, relative chirality populations of skyrmions along with the 2D lattice phase, and the number and location of lattice defects could allow the creation of designer skyrmion liquids or crystals for utilization in neuromorphic computing. Additionally, thermal excitations can be advantageous for reservoir or stochastic computing. We demonstrate a mechanism to tune the rate and ratio of thermal fluctuations by directly influencing the energy barrier between magnetic chiral states. In this context, chirality‐based topological logic gates might be a reality. Our results further emphasize the promise of vdW materials as a platform for both fundamental research and the next‐generation of chiral spintronics applications. The guidelines demonstrated in our work can be promptly adapted to a broad range of other magnets opening several horizons in the exploitation of chirality‐based selectivity of transport properties. This is the natural next step in the creation of energy‐efficient devices, where chirality actively participates in the functionalities.

## 4 Experimental Section

### Lorentz Transmission Electron Microscopy

The samples used in this study were made by dry‐exfoliating CrBr_3_ flakes from a bulk crystal onto a silicon nitride TEM membrane. The flakes were then capped with 3 nm of sputtered Pt to avoid sample degradation and charging in the TEM. The cryo‐LTEM measurements were performed on a JEOL JEM‐2100F TEM with a 200 kV accelerating voltage using a Gatan double‐tilt liquid helium holder. Imaging was performed in Lorentz mode, all LTEM images shown in the manuscript were taken in an underfocus condition at a defocus length of ‐1 mm. The defocus length of the microscope was calibrated through the acquisition of a through focal series of an amorphous carbon gird. In Lorentz mode, the objective lens could be excited to control the magnetic field applied to the sample. The calibration of the objective lens strength to the applied field was performed using a hall probe sensor (Allegro microsystems A1326) mounted on a hummingbird biasing holder. An input voltage of 5V was applied to the hall probe sensor and the change in the output voltage, which was coupled with the change in applied magnetic field, was measured as a function of the current applied to the objective lens. The applied magnetic field was perpendicular to the un‐tilted sample, and a in‐plane magnetic field could be introduced by tilting the sample. All images shown in the manuscript were taken at a temperature of 13 K so as to isolate the effect of the magnetic field. The in‐plane magnetic induction images for the different states were reconstructed using the single image transport of intensity method from the open‐source PyLorentz software.^[^ ^50^ ^]^

### Micromagnetic Simulations

Micromagnetic simulations were performed using MuMax3.^[^ ^54^ ^]^ The material parameters used were as follows: *M*_*s*_ = 270 kAm^−1^, *K*_*u*_ = 86 kJm^−3^ (along the out‐of‐plane direction), *A*_*ex*_ = 1e‐12 Jm^−1^, and D = 0.15 × 10^−3^ Jm^−2^, as in reference.^[^ ^32^ ^]^ Simulations were run for a 250 × 250 × 50 grid with cell sizes of 3.5 × 3.5 × 4 nm. A field cooling procedure was simulated by starting from a randomized initial magnetization at 1400 K. The temperature was then decreased to 1 K in steps of 40 K by running the LLG equation for 5 ns at each temperature step with a 50 mT out‐of‐plane field. The transition to type‐II bubbles was then induced by introducing an “effective tilt” in the magnetic field so as to replicate the experimental procedure. As such, the magnetic field components were defined by the equations *B*_*ip*_ = 50sin (θ) and *B*_*oop*_ = 50cos (θ) mT, where θ started at 0 as was increased in steps of 1°. The LLG equation was run at each angle step for 5 ns. The energy density contributions shown in Figure 1k were calculated using MuMax3. The theoretical phase diagram was generated by simulating the field cooling of the sample at 50 mT then subsequently varying the out‐of‐plane field and introducing an “effective tilt” as above for each out‐of‐plane field value.

### Optical Characterization

The optical response of the CrBr_3_ crystals used for this paper was evaluated by Raman and photoluminescence spectroscopy as shown Figure S1c,d (Supporting Information). The responses shown were consistent with previous reports.^[^ ^63^, ^64^, ^65^ ^]^ Raman scattering and photoluminescence spectra were measured in a microscopic confocal configuration at cryogenic conditions (1.8 K). The sample was cooled down by He exchange gas in a dry closed‐cycle cryostat. The x‐y‐z piezo positioners were used to align the sample under a microscope objective, which focused the laser down to a spot of 1 micron diameter and collimated the optical signal from the sample. A set of optical filters was used to remove the laser Rayleigh scattering. The optical signal from the sample was spectrally dispersed by a 75 cm spectrometer equipped with a charge‐coupled device camera for detection.

## Conflict of Interest

The authors declare no conflict of interest.

## Author Contributions

J.F., Y.L., and H.S. contributed equally to this work. J.F., Y.L., E.J.G.S., and C.P. conceived the project. J.F., Y.L., and C.P. performed low‐temperature LTEM measurements and analyzed the data. S.G., M.G., Z.C., M.S., K.S.N., and M.K. provided the samples and conducted optical microscopy characterization. H.S. and E.J.G.S. performed the micromagnetic simulations. J.F., Y.L., H.S., E.J.G.S., and C.P. wrote the manuscript with inputs from all co‐authors. All co‐authors contributed to this work, read the manuscript, discussed the results, and agreed on the included contents.

## Supporting information

Supporting Information

Supporting Information

Supplemental Video 1

Supplemental Video 2

Supplemental Video 3

Supplemental Video 4

Supplemental Video 5

Supplemental Video 6

Supplemental Video 7

Supplemental Video 8

## Data Availability

The data that support the findings of this study are available from the corresponding author upon reasonable request.
