# Supplementary material for: Observation of Topological Chirality Switching Induced Freezing of a Skyrmion Crystal
Source: Adv Mater. 2025 Oct 28;38(9):e13067. doi: 10.1002/adma.202513067 (PMC12902592; doi:10.1002/adma.202513067)
Supplement: Supplementary file 1 — Supporting Information [file ADMA-38-e13067-s009.pdf]

ADVANCED MATERIALS

Supporting Information

for *Adv. Mater.*, DOI 10.1002/adma.202513067

Observation of Topological Chirality Switching Induced Freezing of a Skyrmion Crystal

John Fullerton, Yue Li*, Harshvardhan Solanki, Sergey Grebenchuk, Magdalena Grzeszczyk, Zhaolong Chen, Makars Šiškins, Kostya S. Novoselov, Maciej Koperski, Elton J. G. Santos* and Charudatta Phatak**

Supplementary Information for "Observation of topological chirality switching induced freezing of a skyrmion crystal"

John Fullerton[1*†], Yue Li[1*†], Harshvardhan Solanki[2†], Sergey Grebenchuk[3,4], Magdalena Grzeszczyk[3], Zhaolong Chen[3,4,5], Makars Šiškins[3], Kostya S. Novoselov[3,4], Maciej Koperski[3,4], Elton J. G. Santos[2,6*], Charudatta Phatak[1,7*]

[1]Materials Science Division, Argonne National Laboratory, Lemont, 60439, IL, United States of America.
[2]Institute for Condensed Matter and Complex Systems, School of Physics and Astronomy, The University of Edinburgh, Edinburgh, EH9 3FD, United Kingdom.
[3]Institute For Functional Intelligent Materials, National University of Singapore, Singapore, 117544, Singapore.
[4]Department of Materials Science and Engineering, National University of Singapore, Singapore, 117544, Singapore.
[5]Donostia International Physics Center — DIPC, Donostia-San Sebastian, 20018, Spain.
[6]Department of Materials Science and Engineering, Northwestern University, Evanston, 60208, IL, United States of America.

*Corresponding author(s). E-mail(s): jfullerton@anl.gov; yue.li@anl.gov; esantos@ed.ac.uk; cd@anl.gov;
†These authors contributed equally to this work.

S1 Sample Characterization

Figure S1a shows an in-focus TEM image of the $CrBr_3$ flake used for the cryo-LTEM experiments shown in the main text. Arrows are overlaid in Figure S1a to denote the directions of the a and b crystal axes and the projected direction of the in-plane

magnetic field. The sample is tilted such that the in-plane field is projected $\approx 15^\circ$ off the crystallographic b axis. Figure S1b shows an electron diffraction pattern of the sample to verify the crystal structure. This is further validated by Raman and Photoluminescence spectroscopy performed on the $CrBr_3$ crystal, as shown in Figures S1c and S1d. Both spectra align with previously observed findings for $CrBr_3$ [1].

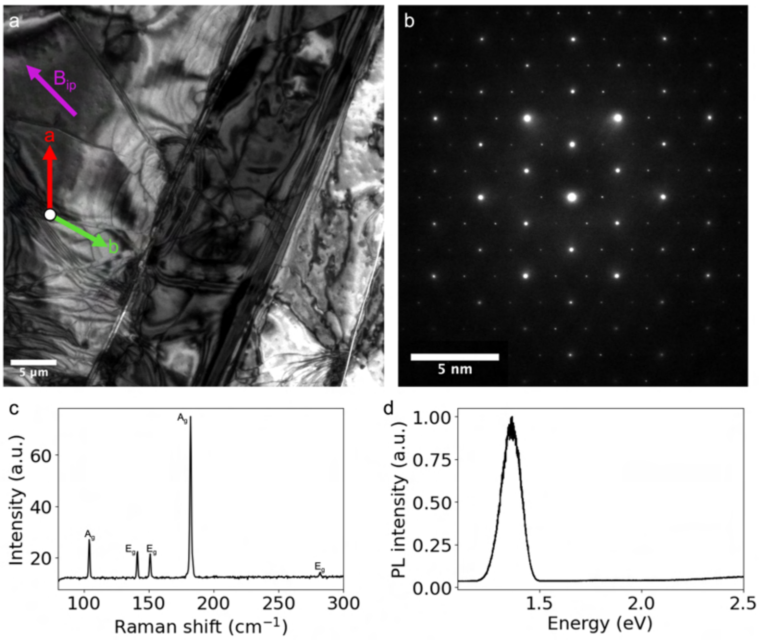

Fig. S1 TEM imaging (a) In-focus TEM image of the sample with arrows to denote the directions of the a and b crystal axes, and the projected direction of the in-plane mangetic field (B_{ip}). (b) Electron diffraction pattern. (c) Raman spectrum of $CrBr_3$, excited by a continuous wave laser at 2.33 eV. (d) Photoluminescence spectrum excited by a laser at 2.67 eV.

S2 Cryo-Lorentz TEM imaging of magnetic bubbles in $CrBr_3$

S2.1 Types of magnetic bubbles

Figure S2a shows depictions of the types of magnetic bubbles in $CrBr_3$. Field cooling with a purely out-of-plane magnetic field stabilizes Bloch bubbles which can show either clockwise or anti-clockwise circulations in the domain wall. If a strong enough in-plane magnetic field is applied, the magnetization in the domain wall of the bubble aligns to the direction of that field. Figure S2b shows simulated LTEM images of each bubble in part a. Experimental LTEM images of Bloch and type-II bubble lattices are shown in Figures S2c and S2e, respectively, along with a schematic depiciton of the

experimental setup in each case. Figures S2d and S2f show schematics of the magnetic energy landscape as it is reshaped by an in-plane magnetic field.

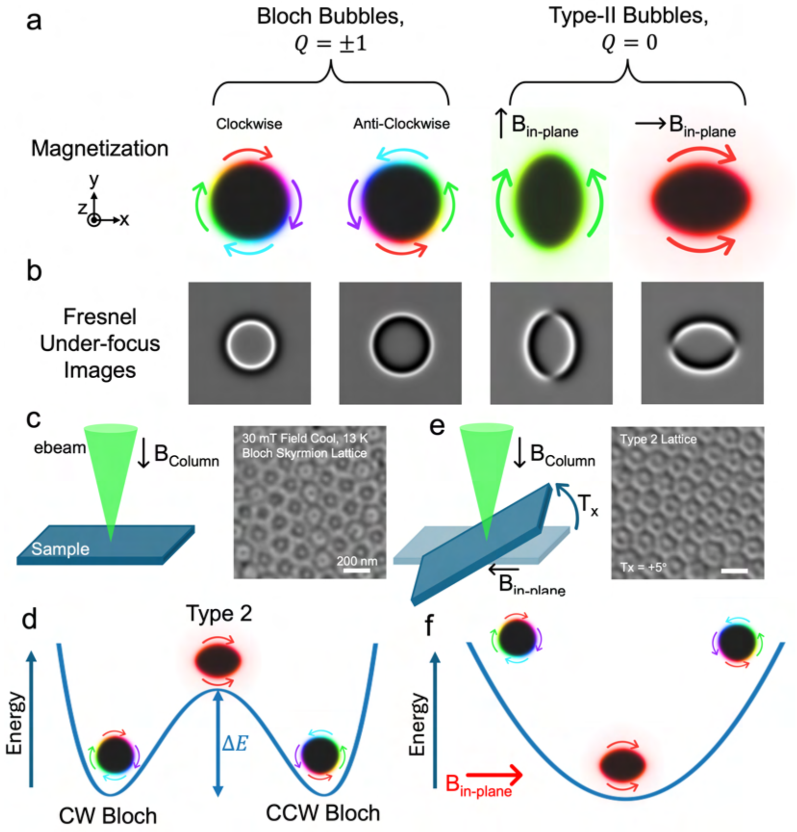

Fig. S2 LTEM contrast of magnetic bubbles (a) Depictions of bubble types seen in $CrBr_3$, from left to right, Bloch bubbles with clockwise and anti-clockwise domain walls, type-II bubbles with fields along orthogonal in-plane directions. (b) Simulated LTEM images of each bubble. (c, d) Depiction of LTEM set-up, image of bubble lattice (c) and schematic of magnetic energy landscape (d) at no tilt. (e, f) Depiction of LTEM set-up, image of bubble lattice (e) and schematic of magnetic energy landscape (f) with a tilt that induces an in-plane field.

S2.2 Experimental application of an in-plane magnetic field

Figure S3a shows the experimental procedure where the sample was repeatably tilted to create an in-plane field component. This is shown experimentally in Figure S3b, where applying and removing and in-plane magnetic field to a Bloch bubble lattice leads to switching of magnetic chirality. By tracking the relative populations of each bubble chirality (Figure S3c), we see that there is always a higher proportion of black (right-handed) bubbles. The in-plane field also has an ordering effect on the lattice

as shown by the decrease in lattice defect density and change in the hexatic order parameter (Figure S3d and S3e, respectively).

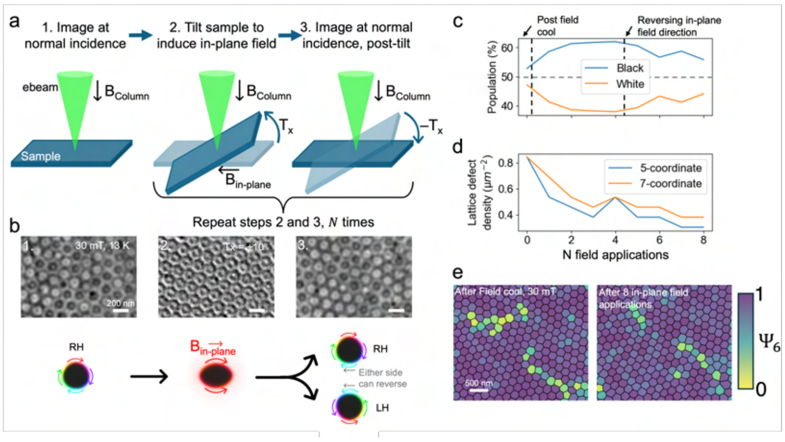

Fig. S3 Effect of successive in-plane field applications. (a) Experimental procedure where the sample was tilted and imaged at normal incidence N times. (b) Example LTEM images and depictions of the effect of a right handed bubble of the procedure shown in part a. (c, d) The relative bubble chirality populations and lattice defect density after each in-plane field application. (e) Voronoi tesselation of the Bloch bubble lattice after field cooling (left) and after 8 applications of an in-plane field.

If we tilt the sample to an intermediate angle, we can minimize the energy barrier between bubble types (Figures S4a and S4b). This allows for spontaneous chirality switching events to occur (Figure S4c). These switching events are highlighted as dark and bright spots in Figure S4d by subtracting consecutive video frames from each other.

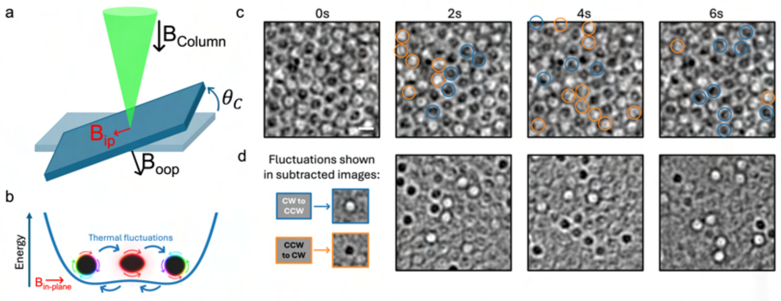

Fig. S4 Inducing thermal fluctuations with an in-plane field. (a, b) Schematic of the LTEM set-up and magnetic energy landscape with an intermediate tilt angle (c) A series of LTEM images with spontaneous chirality switching. (d) Subtracted images of the frames shown in part c, highlighting the chirality switching events.

S2.3 Pairwise spin correlation

To ascertain whether there are interactions between magnetic bubbles that effect the bubble chirality switching events, we calculate the pairwise spin correlation function. The pairwise spin correlation across the bubble lattice is defined as $C = \frac{1}{N}\sum_{i=1}^{N}(S_i \cdot S_j)$, where N is the number of spins, and S_i and S_j are the bubble chiralities at sites i and j [2, 3]. If two bubbles at sites i and j have the same chirality, then $C = 1$, and if they have opposite chirality then $C = -1$. In Figure S5 we consider the pairwise spin correlation for up to the fourth nearest neighbor of a bubble (centered at bubble 21 in S5a, also shown in the main text in Figure 2e). In Figure S5b we plot the pairwise spin correlation over time for each nearest neighbor (left) and also the time averaged correlation (right). Over time, we see random fluctuations in the correlation function for each neighbor which are due to the chirality switching events of each bubble. If we average these over the whole time series, the pairwise correlation function for each nearest neighbor becomes zero. Hence indicating that there are chirality switching events are not strongly influenced by interactions between bubbles.

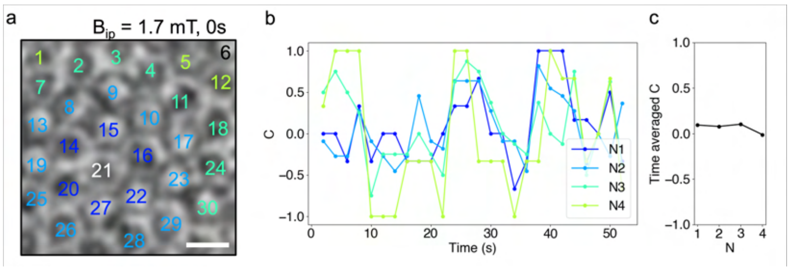

Fig. S5 Pairwise spin correlation of bubble chirality switching. (a) LTEM image of 30 bubbles, all numbered and labeled to show up to the fourth nearest neighbor of bubble 21. (b) The averaged spin correlations for each nearest neighbor as a function of time (left) and averaged over the whole time series (right).

S2.4 Time-dependent skyrmionic lattice order at low field

In Figure S6 we describe the evolution of a skyrmion lattice over time at a given applied field. We initially field-cooled the sample at $B_{oop} = 30$ mT, then, as opposed to the the main text Figure 4, we do not increase the field magnitude. Figures S6a-b we show Voronoi tessellations of a Bloch bubble lattice immediately after field cooling at 30 mT (Figure S6a) and after 12 mins (Figure S6b) while the field and temperature are kept constant at $B_{oop} = 30$ mT, $B_{ip} = 0.32$ mT and 13 K, respectively. The tessellations are colored by the hexatic order parameter. In Figure S6a, we observe three hexagonal lattice grains, which show up as three separate hexagonal peaks in the FFT. Over the course of 12 minutes (Figure S6b), we see the motion and diffusion of defects and a gradual reshuffling of the lattice. Additionally, the three peaks in the FFT blur together, but do not show a single lattice arrangement. Additionally, we observe an exponential decay in the orientational correlation function for both the initial and final images (Figure S6c), indicating that the system remains in the liquid

phase at low field. Hence, we do not reach an ordered state in this time period and there is no significant reduction in the number of lattice defect sites.

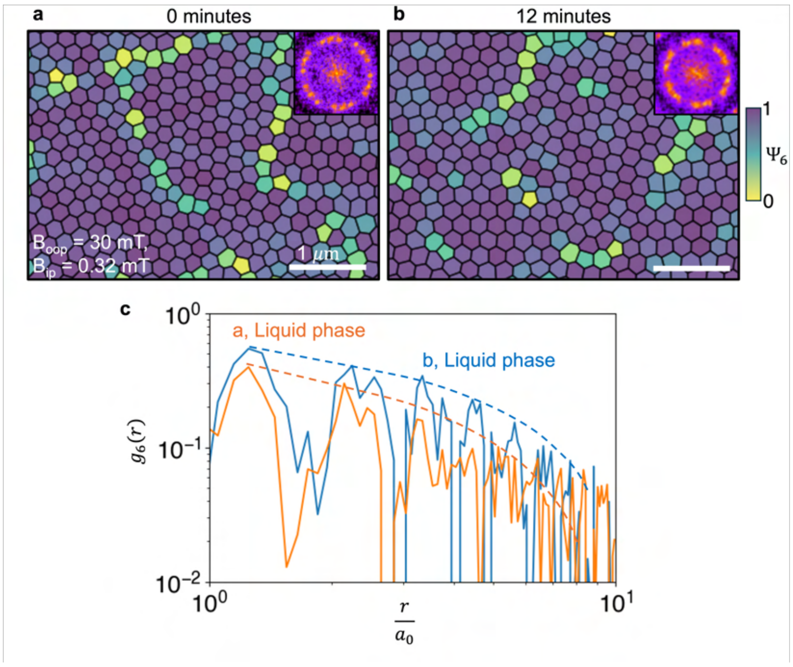

Fig. S6 Lattice order and chirality populations over time. (a, b) Voronoi tesselation of the Bloch bubble lattice over time, with FFTs of each lattice shown in the top right hand corner. (c) Orientational correlation function for the skyrmionic bubble lattices at 0 minutes (blue) and after 12 minutes (orange) for the lattices shown in a and b.

S2.5 Lattice ordering and bubble size with out-of-plane field

Figure S7 shows the effect of applying an out-of-plane magnetic field to a Bloch bubble lattice on the overall lattice order and the size of the bubbles. As expected, we observe that the bubbles shrink with increasing out-of-plane field, while the overall hexatic order parameter decreases.

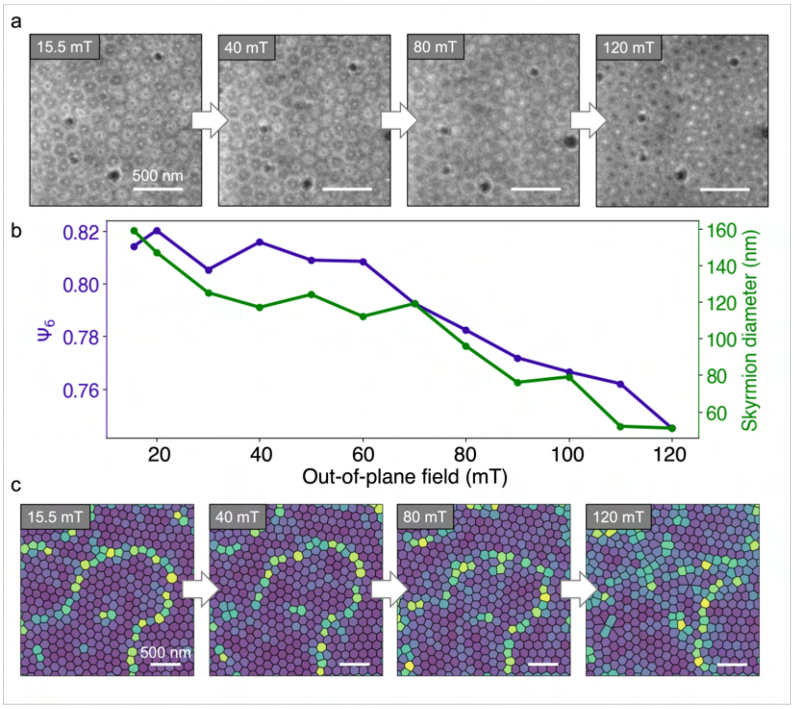

Fig. S7 Lattice order vs out-of-plane field (a) LTEM images of the Bloch bubble lattice with increasing out-of-plane field. (b) Ψ_6 parameter (blue) and skyrmion diameter (green) vs out-of-plane field. (c) Voronoi tesselation of the Bloch bubble lattice with increasing out-of-plane field.

S2.6 Anisotropic skyrmion diffusion with an in-plane field

Figure S8 shows the averaged mean-squared displacement across the skyrmionic bubble lattice in the presence of an in-plane magnetic field (the field values are B_{oop} = 70 mT and B_{ip} = -2.3 mT). The dataset analyzed here is discussed in the main text in figure 4 and is shown in the supplementary videos S7 and S8. Here, we observe a clear anisotropic diffusion along the direction of the in-plane magnetic field (red line in Figure S8) [4].

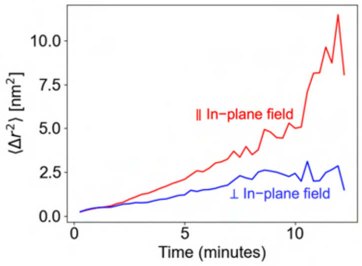

Fig. S8 Experimental averaged mean-squared displacement of a skyrmionic bubble lattice as a function of time under the application of an in-plane magnetic field. The red line shows the displacement along the in-plane field direction and the blue line shows the displacement perpendicular to the in-plane field direction.

S3 Micromagnetic simulations

Micromagnetic simulations of CrBr3 were conducted to support the experimental analy- sis using MuMax3 [5–7]. The material parameters used for the simulations followed from precalculated values by Grebunchek et al [1].

$$l_{ex} = \sqrt{\frac{2A}{\mu_0 M_s^2}} \quad (1)$$

Equation (1) was used to determine that the exchange length of the material was around 6.11 nm. The simulation was then setup by creating a 250 × 250 × 50 grid with each cell having 3.5 × 3.5 × 4 nm dimensions, ensuring that the dimensions of the cells are less than the exchange length. Starting from a randomized spin state, the set-up was then field cooled from 1400 K to 1 K in steps of 40 K by running the LLG equation at each temperature step for 5 ns with a constant out-of-plane (OOP) field. The OOP field value was set to 50 mT for most simulations, except those where the dependence of OOP and IP field was investigated. At this value of the OOP field the system stabilized a Bloch skyrmion lattice with a mixed ratio of right-handed (RH) and left-handed (LH) skyrmions of polarity opposite to that of the applied OOP field, although with a preference for RH skyrmions due to weak DMI. It was also shown that the weak DMI is the primary cause of remnant Neel-like behaviour in the Bloch skyrmions as the Neel caps on the top and bottom surface are not exact cancellations of each other when averaged (See Figure S9).

The final field-cooled state was then used to apply an in-plane field in positive X, such that the total magnitude of the applied external field was kept constant by simultaneously reducing the OOP component according to equations $B_x = 50\sin(\theta)$ and $B_z = 50\cos(\theta)$) where θ is the tilt angle in radians. This simulates the "tilting" as performed in the experiments, with a constant 50 mT magnitude field with tilt angle going from 0° to 90° in increments of 1°, then brought back to 0°. The LLG equation was run at each angle increment for 5 ns and the system was subsequently relaxed to ground state. This procedure showed excellent agreement with the experimental results

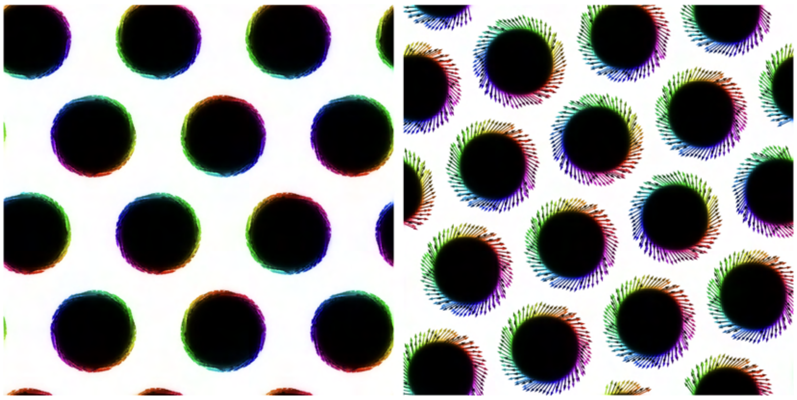

Fig. S9 Example layer-averaged magnetisation states of the field-cooled system without (left) DMI and with (right) DMI

since type-II bubbles formed at higher values of in-plane field, and then, on reducing the in-plane component to 0 mT, skyrmions were re-obtained. The final state shows that the skyrmions that were originally LH have retained some type-II characteristics even in the absence of the in-plane field (See Figure S9). This is likely due to the weak DMI, external torque, and Bloch-point arguments as discussed in the main text. Further testing on the final state after increasing the IP field in the +X direction showed that the skyrmions are stable even in the absence of all fields after the tilting procedure, as they persist (and even remove all type-II bubble character) when the external field is removed. This is expected since in the absence of fields skyrmions lie in metastable local minima on the energy surface and type-II bubbles are unstable local maxima.

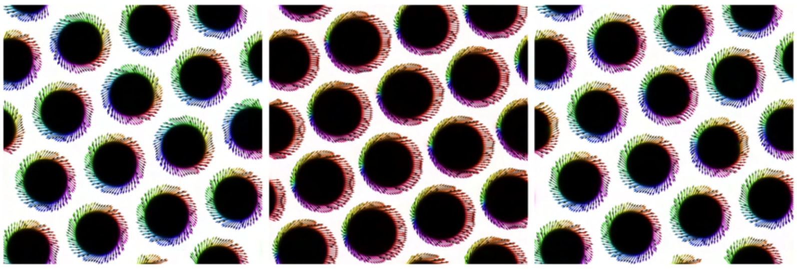

Fig. S10 Snapshots of the sample tilting: before the process begins (left), at maximum tilt (middle), and after re-leveling (right).

The IP field was then applied in the -X direction on the same system after a positive tilt, creating an effective hysteresis loop of the IP field. The type-II bubbles are still formed with increasing IP field as expected, but the skyrmions that retained type-II characteristics initially have now broken into a mix of RH states and type-II bubbles in the predominantly -X direction (See Figure S12).

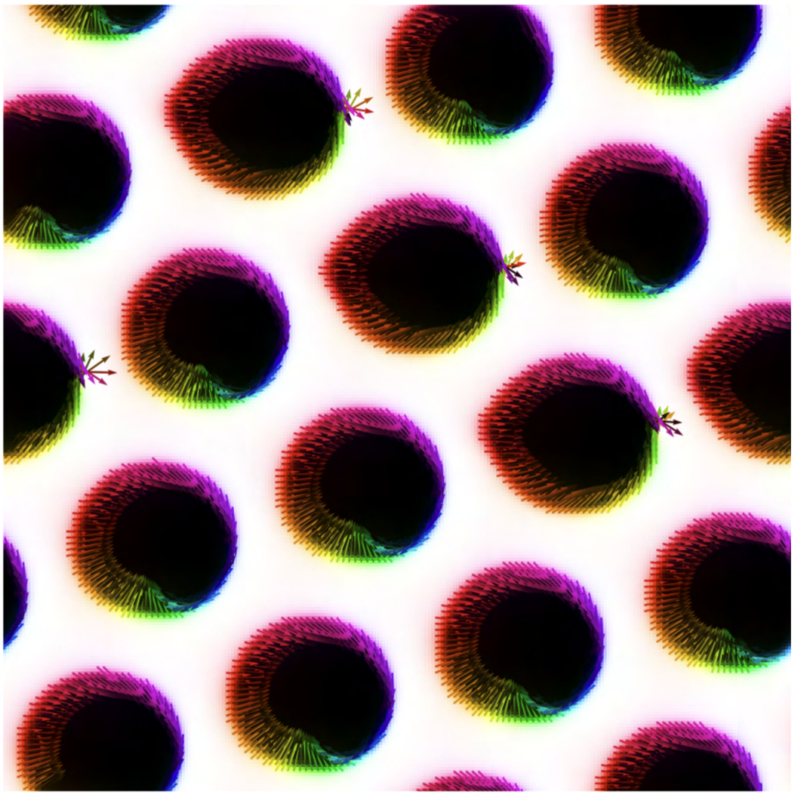

Fig. S11 Layer Dependence of the "full-tilted" state to observe the imprint of the RH and LH skyrmions in the type-II bubbles.

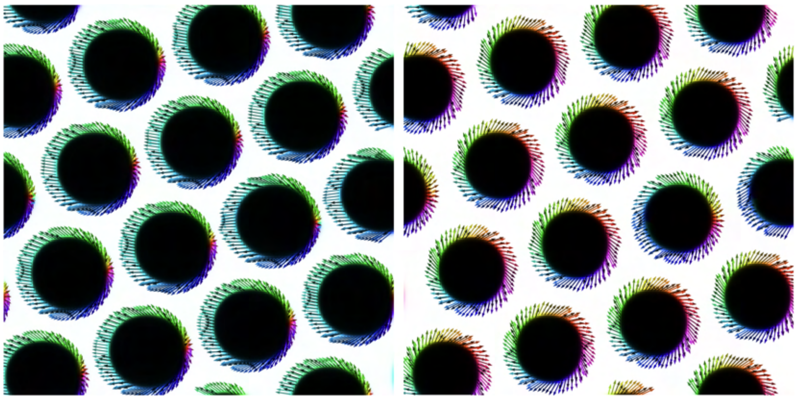

Fig. S12 Result of tilting the same system in the opposite X direction. We see that some of the LH skyrmions retain their type-II characteristics similar to the first tilt, but the rest have flipped to RH chirality.

Conducting the same "tilting" simulations on a system without DMI showed that type-II bubbles were formed at much larger values of the IP field, at around effective

tilt values of 33°-34°, and that the type-II behavior persists in the system even on removal of the IP field. This provides strong support for the physical presence of DMI in the experimental samples since they tend to return to a skyrmion lattice under a purely OOP external field (See Figure S13). Significantly, we observe that Bloch bubbles of both chiralities switch to type-II bubbles at the same field value. Showing, that the presence of DMI is vital in biasing the chirality switching behavior towards a given chirality.

Testing various random initial configurations with and without the presence of DMI also showed that there is a preferential direction for the skyrmion chirality when DMI is present in the material, and this also supports the physical presence of DMI since the experimental samples observe a slight preference for RH Bloch skyrmions over LH (See Figure S14).

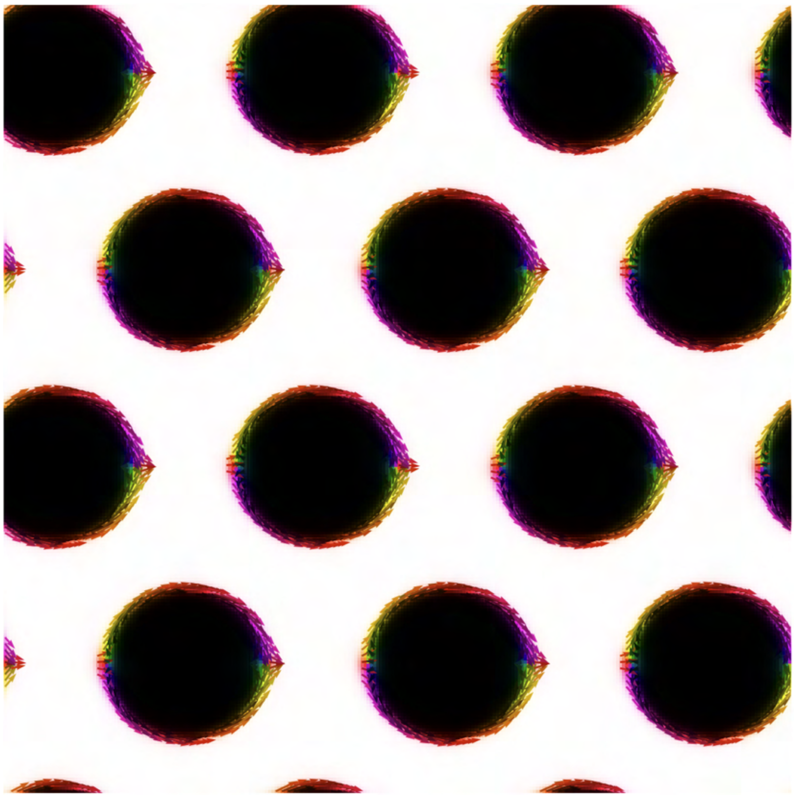

Fig. S13 Final state of tilting simulations on a system excluding DMI. It can be seen clearly that even after re-levelling the system all skyrmions have retained their type-II bubble characteristics.

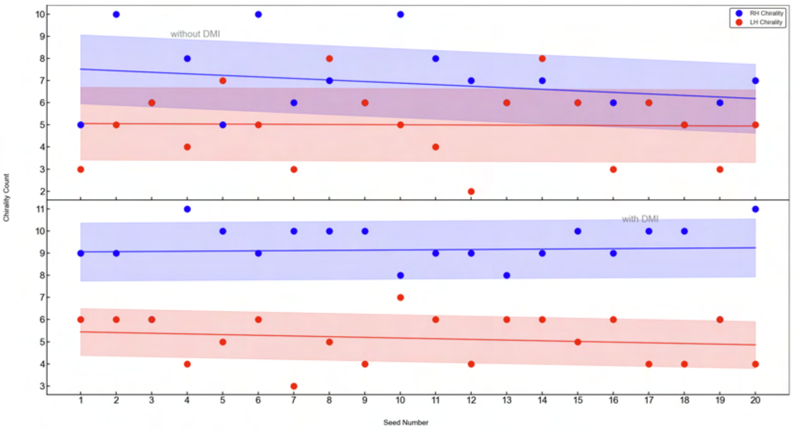

Fig. S14 Chirality count (RH or LH count) against seed number for 20 seeds. On top are the simulations conducted excluding DMI, and on the bottom are those with DMI. There is a clear trend for RH skyrmions to dominate in the presence of DMI.

References

[1] Grebenchuk, S., McKeever, C., Grzeszczyk, M., Chen, Z., Šiškins, M., McCray, A.R.C., Li, Y., Petford-Long, A.K., Phatak, C.M., Ruihuan, D., Zheng, L., Novoselov, K.S., Santos, E.J.G., Koperski, M.: Topological spin textures in an insulating van der Waals ferromagnet. Advanced Materials **36**(24), 2311949 (2024) https://doi.org/10.1002/adma.202311949

[2] Chioar, I.A., Canals, B., Lacour, D., Hehn, M., Santos Burgos, B., ş, T.O., Locatelli, A., Montaigne, F., Rougemaille, N.: Kinetic pathways to the magnetic charge crystal in artificial dipolar spin ice. Phys. Rev. B **90**, 220407 (2014) https://doi.org/10.1103/PhysRevB.90.220407

[3] Li, Y., Barrows, F., McCray, A.R.C., Cote, T., Friedman, D., Divan, R.N.S., Petford-Long, A.K., Phatak, C.: Geometric control of emergent antiferromagnetic order in coupled artificial spin ices. Cell Reports Physical Science **3**(4), 100846 (2022) https://doi.org/10.1016/j.xcrp.2022.100846

[4] Kerber, N., Weißenhofer, M., Raab, K., Litzius, K., Zázvorka, J., Nowak, U., Kläui, M.: Anisotropic skyrmion diffusion controlled by magnetic-field-induced symmetry breaking. Physical Review Applied **15**(4), 044029 (2021) https://doi.org/10.1103/PhysRevApplied.15.044029

[5] Vansteenkiste, A., Leliaert, J., Dvornik, M., Helsen, M., Garcia-Sanchez, F., Van Waeyenberge, B.: The design and verification of MuMax3. AIP Advances **4**(10), 107133 (2014) https://doi.org/10.1063/1.4899186 https://pubs.aip.org/aip/adv/article-pdf/doi/10.1063/1.4899186/12878560/107133_1_online.pdf

[6] Mulkers, J., Van Waeyenberge, B., ć, M.V.: Effects of spatially engineered Dzyaloshinskii-Moriya interaction in ferromagnetic films. Phys. Rev. B **95**, 144401 (2017) https://doi.org/10.1103/PhysRevB.95.144401

[7] Leliaert, J., Mulkers, J., De Clercq, J., Coene, A., Dvornik, M., Van Waeyenberge, B.: Adaptively time stepping the stochastic landau-lifshitz-gilbert equation at nonzero temperature: Implementation and validation in mumax3. AIP Advances **7**(12), 125010 (2017) https://doi.org/10.1063/1.5003957 https://pubs.aip.org/aip/adv/article-pdf/doi/10.1063/1.5003957/19733887/125010_1_online.pdf
