# Supplementary material for: Observation of Topological Chirality Switching Induced Freezing of a Skyrmion Crystal
Source: Adv Mater. 2025 Oct 28;38(9):e13067. doi: 10.1002/adma.202513067 (PMC12902592; doi:10.1002/adma.202513067)
Supplement: Supplementary file 2 — Supporting Information [file ADMA-38-e13067-s005.pdf]

ADVANCED MATERIALS

Supporting Information

for *Adv. Mater.*, DOI 10.1002/adma.202513067

Observation of Topological Chirality Switching Induced Freezing of a Skyrmion Crystal

John Fullerton, Yue Li*, Harshvardhan Solanki, Sergey Grebenchuk, Magdalena Grzeszczyk, Zhaolong Chen, Makars Šiškins, Kostya S. Novoselov, Maciej Koperski, Elton J. G. Santos* and Charudatta Phatak**

Guide for Supplementary information

1 Supplementary Information

A PDF file containing further experimental data and details on micromagnetic simulations to support the main text.

2 Supplementary Video 1

The video shows the spontaneous chirality switching at field values of $B_{oop} = 300$ G and $B_{ip} = 11$ G.

3 Supplementary Video 2

The video shows the spontaneous chirality switching at field values of $B_{oop} = 500$ G and $B_{ip} = 15$ G, used to extract the data for figures 2b and 2e.

4 Supplementary Video 3

The video shows the spontaneous chirality switching at field values of $B_{oop} = 500$ G and $B_{ip} = 18$ G, used to extract the data for figures 2c and 2f.

5 Supplementary Video 4

The video shows the spontaneous chirality switching at field values of $B_{oop} = 700$ G and $B_{ip} = 18$ G.

6 Supplementary Video 5

The video shows the spontaneous chirality switching at field values of $B_{oop} = 900$ G and $B_{ip} = 20$ G.

7 Supplementary Video 6

The video shows the bubble lattice diffusion and motion at field values of $B_{oop} = 300$ G and $B_{ip} = 3$ G, used to extract data for figures 4a, 4b, 4e and 4f.

8 Supplementary Video 7

The video shows the bubble lattice diffusion, motion and the spontaneous chirality switching at field values of $B_{oop} = 700$ G and $B_{ip} = 23$ G, used to extract data for figures 4c, 4d, 4e and 4f.

9 Supplementary Video 8

The video shows the Voronoi tessellations extracted from supplementary video 7. Two frames of which were used for figures 4c and 4d.
